# Leadless Versus Transvenous Dual‐Chamber Pacemakers: Real‐World Evidence From AVEIR DR Coverage With Evidence Development Study

**DOI:** 10.1111/jce.70255

**Published:** 2026-01-16

**Authors:** Monica Lo, Anish K. Amin, Marcin Kowalski, Anne Kroman, Dilip Mathew, Valay Parikh, Stanislav Weiner, Jennifer M. Joseph, Leonard Ganz, Mohammed Mortada, AVEIR DR Study Group/Consortium

**Affiliations:** ^1^ Arkansas Heart Hospital Little Rock Arkansas USA; ^2^ OhioHealth Heart and Vascular Physicians Columbus Ohio USA; ^3^ Zucker School of Medicine at Hofstra/Northwell Hempstead New York USA; ^4^ Staten Island University Hospital, Northwell Health Staten Island New York USA; ^5^ Medical University of South Carolina Charleston South Carolina USA; ^6^ Sarasota Memorial Hospital Sarasota Florida USA; ^7^ Christus Health Tyler Texas USA; ^8^ Abbott Irvine California USA; ^9^ Abbott Sylmar California USA; ^10^ Aurora St. Luke's Medical Center Milwaukee Wisconsin USA; ^11^ Wake Forest University School of Medicine Winston‐Salem North Carolina USA

**Keywords:** AVEIR DR, leadless pacemakers, outcomes, reinterventions, safety, transvenous pacemakers

## Abstract

**Background:**

AVEIR DR, an industry‐first dual‐chamber leadless pacemaker (LP), provides continuous atrioventricular synchrony through implant‐to‐implant (i2i) communication between atrial and ventricular LP devices. It is important to evaluate the early real‐world comparative safety of AVEIR DR LP.

**Objective:**

To compare complications and mortality between AVEIR DR LP and dual‐chamber transvenous pacemakers (TP).

**Methods:**

De novo LP and TP patients were identified in Medicare Fee‐for‐Service claims (October 2023 and December 2024). Outcomes were 30‐day and 6‐month complications, reinterventions, heart failure hospitalizations, and all‐cause mortality. Comparisons were adjusted for demographics, comorbidities, and hospital encounter characteristics.

**Results:**

Compared to TP (*N* = 77 422, age = 79.6 ± 7.5), LP patients (*N* = 759, age = 78.5 ± 7.8) had a higher comorbidity index, more dialysis dependence, end‐stage renal disease, and atrial fibrillation. Adjusted 30‐day overall complications (7.9% vs. 9.2%; odds ratio [OR] 0.85, *p* = 0.36) and mortality (1.8% vs. 1.5%; hazard ratio [HR] 1.21, *p* = 0.47) were comparable, while device‐related complications (2.0% vs. 3.9%; OR 0.50, *p* < 0.01) were lower with LP. Adjusted 6‐month overall complications (4.1% vs. 6.9%; HR 0.59, *p* < 0.01), device‐related complications (2.8% vs. 5.9%; HR 0.48, *p* < 0.01), and device reinterventions (2.1% vs. 4.3%; HR 0.49, *p* < 0.01) were reduced with LP, with no difference in mortality (6.6% vs. 5.6%; HR 1.18, *p* = 0.43) and heart failure hospitalizations (3.8% vs. 4.1%; HR 0.91, *p* = 0.65).

**Conclusion:**

Despite a higher comorbidity burden, AVEIR DR LP had significantly fewer device‐related complications, overall 6‐month complications, and device reinterventions compared to TP, with similar overall 30‐day complications, mortality, and heart failure hospitalizations.

**Abbreviations**
ALP: atrial leadless cardiac pacemaker
AV: atrioventricular
CED: coverage with evidence development
CIED: Cardiac Implantable Electronic Device
CMS: The Centers for Medicare & Medicaid Services
CRT: cardiac resynchronization therapy
DVT: deep vein thrombosis
FDA: Food and Drug Administration
FFS: Fee‐for‐Service
ICD: implantable cardioverter defibrillator
i2i: implant‐to‐implant
LP: leadless cardiac pacemaker
MBSF: Master Beneficiary Summary Files
RA: right atrium
RV: right ventricle
TP: dual‐chamber transvenous pacemaker
VLP: ventricular leadless cardiac pacemaker

## 1 Introduction

Leadless pacemakers (LPs) are a recent technology developed to obviate lead and pocket complications related to traditional transvenous pacemakers (TP) [1]. The AVEIR DR (Abbott, Chicago, IL) dual‐chamber leadless pacemaker system is a novel LP system which includes a ventricular LP (VLP) and a smaller atrial LP (ALP). The system senses and paces in both the right atrium (RA) and right ventricle (RV), using implant‐to‐implant (i2i) communication between the two LPs to coordinate timing and achieve true, continuous atrioventricular (AV) synchrony [2].

The safety, electrical performance, i2i communication, and battery longevity of AVEIR DR LP were demonstrated in a prospective, international clinical trial (AVEIR DR i2i Study, NCT05252702) [3, 4, 5]. Recent findings from a multicenter study evaluating early clinical experience with the AVEIR DR LP system further support its safety and efficacy in real‐world settings [2]. However, comparative real‐world data have been missing.

Centers for Medicare & Medicaid Services (CMS) mandates national coverage determination (NCD) for AVEIR DR LP with a coverage with evidence development (CED) study to compare complication rates and patient outcomes between AVEIR DR LP and dual‐chamber TP patients at various time points up to 2 years. We present early results from the CED comparing 30‐day and 6‐month outcomes between AVEIR DR LP and dual‐chamber TP recipients.

## 2 Methods

### 2.1 Study Design and Data Sources

The AVEIR DR LP CED study is a comparative observational study using the most current available Medicare Fee‐for‐Service (FFS) insurance claims and Abbott device registration data. Medicare data files include institutional claims (inpatient, outpatient), non‐institutional claims (carrier), and Master Beneficiary Summary Files (MBSF) which contain demographics, birth and death dates, Medicare eligibility status, and enrollment dates. Abbott device registration data contains date of birth, sex, device type, implant dates, and implanting facility.

### 2.2 Study Population

The study included two groups of patients implanted with dual‐chamber pacemakers: (1) AVEIR DR LP implanted after CMS approval of the AVEIR DR CED study (October 2023 and December 2024) and (2) dual‐chamber TP implants from all manufacturers as the control group (October 2023 and December 2024).

Patients implanted with an LP or TP in the inpatient setting were identified in Medicare inpatient claims using *International Classification of Diseases, 10th Revision, Procedure Coding System* (Supporting Information S1: Table S1); patients implanted in the outpatient setting were identified in Medicare outpatient claims using Current Procedural Terminology (CPT) Fourth Edition, Healthcare Common Procedure Coding System (HCPCS) (Supporting Information S1: Table S1). Of patients implanted with an LP, those implanted with AVEIR DR LP were then identified by linking Medicare with Abbott device registration data using probabilistic linking [6]. Linking variables included date of birth, sex, device type, implant dates, and implanting facility. The index date for both study groups was the implant procedure date from Medicare claims.

Inclusion criteria required patients to have at least 12 months of continuous Medicare FFS coverage prior to the implant, and coverage for at least 30 days post‐implant or until death if within 30 days. The 12‐month period prior to the implant allows for the collection of clinical characteristics from the claims data, such as comorbidities and a history of relevant procedures. Implants were also required to be de novo; patients who had other Cardiac Implantable Electronic Device (CIED) implants (Supporting Information S1: Table S2) prior to the index date were excluded from the study.

This study was conducted as a retrospective analysis of de‐identified data. The study was granted a full waiver of informed consent and a HIPAA waiver from Western IRB and is registered on ClinicalTrials.gov [7].

### 2.3 Outcome Measures

Primary outcome measures included (1) overall acute complication rate, defined as periprocedural adverse events occurring within 30 days of implant, and (2) all‐cause mortality rate within 30 days of implant. The following acute complications were included in overall acute complication rate: deep vein thrombosis (DVT), pulmonary embolism, thrombosis, embolism, arteriovenous fistula, vascular pseudoaneurysm, pericardial effusion, cardiac perforation, cardiac tamponade, device dislodgement or displacement, infection, hemorrhage, device malfunction, pain, stenosis, pocket complication, postprocedural hematoma, postprocedural hemorrhage, pericarditis, acute myocardial infarction, intraoperative cardiac arrest, bleeding or failure of vascular closure device requiring intervention, hemothorax, and pneumothorax. Complications were identified by the presence of a diagnosis code indicative of a device‐ or procedure‐related complication. Diagnosis codes could appear in any position on the claim: as the primary diagnosis or any of the secondary diagnoses. Post‐implant infections and DVTs were considered complications only if the patient did not have these diagnoses in the 30 days prior to their index implant. See Supporting Information S1: Table S3 for individual acute complication diagnosis codes and operational definitions.

Secondary outcome measures included (1) overall chronic complication rate, defined as postprocedural adverse events occurring within 6 months of implant, (2) device‐related reintervention rate occurring within 6 months of implant, (3) all‐cause mortality rate occurring within 6 months of implant, and (4) heart failure hospitalizations within 6 months of implant. The following chronic complications were included in the overall chronic complication rate: thrombosis, embolism, device malfunction, device dislodgement or displacement, infection, hemorrhage, pain, stenosis, pocket complication, pericarditis, and hemothorax. Chronic complications were identified by the presence of a diagnosis code that indicates a device‐ or procedure‐related adverse event. Diagnosis codes could appear in any position on the claim: as the primary diagnosis or as any of the secondary diagnoses. Device‐related reinterventions were identified by the presence of a procedure code indicating a new pacemaker implant or device replacement with a single‐chamber or dual‐chamber transvenous or LP, device revision, or device explant. Upgrades to cardiac resynchronization therapy (CRT) and implantable cardioverter defibrillator (ICD) devices were also defined as a reintervention. Heart failure hospitalizations were identified by the presence of a primary diagnosis code on an inpatient claim. See Supporting Information S1: Table S4 for individual chronic complication diagnosis codes and operational definitions. See Supporting Information S1: Table S5 for device‐related reintervention procedure codes and operational definitions.

### 2.4 Statistical Analyses

Baseline characteristics obtained from Medicare FFS claims in the 12 months prior to index implant included demographics (age, sex, race/ethnicity, dual eligibility status for Medicare and Medicaid), clinical characteristics (see Supporting Information S1: Table S6 for diagnosis and procedure codes), and healthcare encounter characteristics (number of days from admission to implant and indicator variables for inpatient implant, admission through emergency room, temporary pacing during hospitalization, and weekend implant). Differences in these baseline characteristics were compared between AVEIR DR LP and TP patients using *t*‐tests for continuous variables and *χ* ^2^ tests for categorical variables.

Propensity score overlap weights were used to adjust for differences in baseline characteristics between AVEIR DR LP and TP patients. Overlap weights were calculated from propensity scores based on methods described by Li et al., which emphasize patients with the most overlap in their observed characteristics, providing more precise treatment effects [8]. Another advantage of the overlap weights method over other methods is that it down‐weights extreme values rather than removes them. The acute complication rate was compared between AVEIR DR LP and TP patients with unadjusted and adjusted logistic regression models. The chronic complication rate, heart failure hospitalization rate, and device reintervention rate were compared between the study groups with unadjusted and adjusted Fine‐Gray competing risk models, with death as the competing risk. The 30‐day and 6‐month mortality rates were compared between the study groups with unadjusted and adjusted Cox proportional hazard models. All models account for clustering at the implant hospital. For all Fine‐Gray competing risk and Cox proportional hazard models, patients were censored at the earliest of the following events: (1) after experiencing the outcome, (2) 6 months after implant, (3) end of claims data availability, (4) end of Medicare FFS enrollment, (5) CIED implant, including leadless single‐chamber pacemaker, transvenous single‐chamber pacemaker, dual‐chamber LP, dual‐chamber TP, ICD, cardiac resynchronization therapy pacemaker (CRT‐P), or defibrillator (CRT‐D), (6) leadless device explant, TP system explant, or battery removal/replacement, and (7) death. For all outcomes, events were counted toward the event rates before the patient was censored. All analyses were conducted in SAS Enterprise Guide version 7.15 (SAS Institute Inc.).

## 3 Results

### 3.1 Patient Characteristics

**Figure 1:**
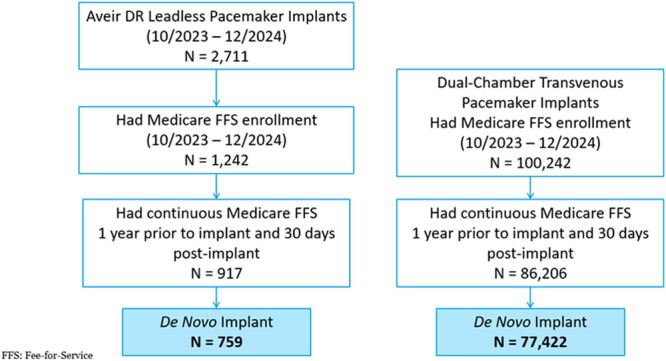
Cohort diagram. Patients were included in the study if they were Medicare beneficiaries implanted with an AVEIR DR leadless pacemaker or a dual‐chamber transvenous pacemaker. Inclusion criteria required patients to have at least 12 months of continuous Medicare Fee‐For‐Service (FFS) coverage prior to the implant, and coverage for at least 30 days post‐implant or until death if within 30 days. Implants were required to be de novo; patients who had other Cardiac Implantable Electronic Device (CIED) implants prior to the index date were excluded from the study.

A total of 102 953 Medicare FFS beneficiaries were identified (AVEIR DR LP, *n* = 2711; TP, *n* = 100 242). After excluding patients who did not have at least 12 months of continuous Medicare FFS coverage prior to implant and 30 days post‐implant or had a prior CIED, there were 759 AVEIR DR LP and 77 422 TP patients included in this analysis (Figure 1). Mean (min, max) follow‐up was 126.1 (1, 180) days for AVEIR DR LP and 144.5 (0, 180) days for TP. Differences in baseline characteristics were observed between the two study groups, as shown in Table 1. Compared to TP, AVEIR DR LP patients were slightly younger (78.5 ± 7.8 vs. 79.6 ± 7.5 years, *p* < 0.01) and more racially diverse. In the AVEIR DR LP group, there were more inpatient implants, though statistically fewer weekend implants or admissions through the emergency room compared to TP.

**Table 1:** Baseline characteristics.

	AVEIR DR (*N* = 759)	Transvenous dual‐chamber pacemaker (*N* = 77 422)	*p* value
Demographic characteristics			
Age, mean (SD), years	78.5 (7.8)	79.6 (7.5)	< 0.01
Female sex	302 (39.8%)	29 301 (37.8%)	< 0.01
Race			< 0.01
White	651 (85.8%)	69 742 (90.1%)	
Black	46 (6.1%)	3183 (4.1%)	
Asian/Hispanic/Native American	18 (2.4%)	1932 (2.5%)	
Other/unknown	44 (5.8%)	2565 (3.3%)	
Dual eligibility	71 (9.4%)	6527 (8.4%)	0.36
Hospital encounter characteristics			
Inpatient implant	437 (57.6%)	41 435 (53.5%)	0.03
Admission to implant, mean (SD), days	1.8 (4.1)	1.3 (2.6)	< 0.01
Weekend implant	< 11	2522 (3.3%)	< 0.01
Admission through emergency room	214 (28.2%)	27 687 (35.8%)	< 0.01
Temporary pacing during hospitalization	43 (5.7%)	5246 (6.8%)	0.23
Clinical characteristics			
Atrial and ventricular arrhythmias			
Atrial fibrillation	440 (58.0%)	41 327 (53.4%)	0.01
Atrial flutter	153 (20.2%)	14 464 (18.7%)	0.30
Ventricular arrhythmia	164 (21.6%)	15 053 (19.4%)	0.13
AV block	470 (61.9%)	47 742 (61.7%)	0.88
Sinus node dysfunction	545 (71.8%)	49 352 (63.7%)	< 0.01
Charlson comorbidity index, mean (SD)	4.4 (3.2)	4.0 (3.0)	< 0.01
Chronic obstructive pulmonary disease	176 (23.2%)	16 583 (21.4%)	0.24
Coronary artery disease	451 (59.4%)	42 251 (54.6%)	< 0.01
Diabetes	323 (42.6%)	30 619 (39.5%)	0.09
Heart failure	286 (37.7%)	31 715 (41.0%)	0.07
Hyperlipidemia	663 (87.4%)	66 328 (85.7%)	0.19
Hypertension	713 (93.9%)	72 317 (93.4%)	0.56
Peripheral vascular disease	172 (22.7%)	16 017 (20.7%)	0.18
Recent infection due to cardiac implants/prosthetic devices	< 11	33 (0.0%)	< 0.01
COVID‐19 within 30 days before implant	20 (2.6%)	1336 (1.7%)	0.06
Prior cardiovascular events and procedures			
Prior coronary artery bypass graft	104 (13.7%)	8796 (11.4%)	0.04
Prior acute myocardial infarction	135 (17.8%)	13 446 (17.4%)	0.76
Prior percutaneous coronary intervention	135 (17.8%)	12 309 (15.9%)	0.16
Concomitant atrial ablation	20 (2.6%)	1806 (2.3%)	0.58
Concomitant TAVR	12 (1.6%)	2071 (2.7%)	0.06
Prior TAVR	11 (1.5%)	1544 (2.0%)	0.26
Renal disease	367 (48.4%)	35 978 (46.5%)	0.30
End‐stage renal disease	50 (6.6%)	972 (1.3%)	< 0.01
Dialysis dependence	48 (6.3%)	846 (1.1%)	< 0.01
Tricuspid valve disease	234 (30.8%)	21 733 (28.1%)	0.09
Tricuspid valve regurgitation	136 (17.9%)	12 097 (15.6%)	0.08

*Note:* To comply with the CMS cell size suppression policy, a cell containing a value of 1–10 cannot be reported directly; therefore, “< 11” is used to display a value of 1–10.

AVEIR DR LP patients had more atrial fibrillation (58.0% vs. 53.4%, *p* = 0.01), sinus node dysfunction (71.8% vs. 63.7%, *p* < 0.01), coronary artery disease (59.4% vs. 54.6%, *p* < 0.01), end‐stage renal disease (6.6% vs. 1.3%, *p* < 0.01), and dialysis dependence (6.3% vs. 1.1%, *p* < 0.01). The higher comorbidities were reflected by the higher Charlson comorbidity index among AVEIR DR LP patients (4.4 ± 3.2 vs. 4.0 ± 3.0, *p* < 0.01).

### 3.2 Acute Complications

Before and after adjustment, there was no significant difference in overall acute complications between AVEIR DR LP and TP (adjusted rate 7.9% vs. 9.2%; odds ratio (OR) 0.85, 95% CI 0.60 to 1.20). The 30‐day time to event graph is shown in the Supporting Information S1: Graph S7. Adjusted and unadjusted overall and individual acute complication rates are shown in Table 2. AVEIR DR LP had significantly lower acute device‐related complications (adjusted rate 2.0% vs. 3.9%, *p* < 0.01; OR 0.50, 95% CI 0.31 to 0.82), which is a 50% risk reduction compared to TP. This is due to AVEIR DR LP having a lower rate of most individual device‐related complications, including a lower rate of dislodgement (adjusted rate 1.0 vs. 1.7, *p* = 0.15) and device malfunction (adjusted rate 0.9 vs. 1.3, *p* = 0.36). AVEIR DR LP had a higher rate of cardiac effusion/perforation (adjusted rate 1.2 vs. 0.6, *p* = 0.047) and procedure‐related complications, including procedure‐related bleeding (adjusted rate 0.8 vs. 0.1, *p* < 0.01).

**Table 2:** Unadjusted and adjusted 30‐day complication rates.

	Unadjusted			Adjusted		
	AVEIR DR (*N* = 759) *n* (%)	Transvenous dual‐ chamber pacemaker (*N* = 77 422) *n* (%)	*p* value	AVEIR DR (*N* = 759) %	Transvenous dual‐chamber pacemaker (*N* = 77 422) %	*p* value
Overall complications	8.0%	8.3%	0.62	7.9%	9.2%	0.36
Individual complications						
Embolism and thrombosis	18 (2.4)	1778 (2.3)	0.89	2.3	2.5	0.77
Deep vein thrombosis	11 (1.5)	859 (1.1)	0.37	1.4	1.2	0.75
Pulmonary embolism	< 11	990 (1.3)	0.82	1.2	1.3	0.76
Thrombosis due to cardiac device	0	39 (0.05)	N/A	0	0.04	N/A
Embolism due to cardiac device	< 11	< 11	< 0.01	0.1	0	N/A
Events at puncture site	< 11	169 (0.2)	< 0.01	1.0	0.5	0.07
Cardiac effusion/perforation	< 11	419 (0.5)	0.02	1.2	0.6	0.047
Device‐related complication	15 (2.0)	2890 (3.7)	0.01	2.0	3.9	< 0.01
Device malfunction	< 11	1076 (1.4)	0.28	0.9	1.3	0.36
Device dislodgement	< 11	1316 (1.7)	0.17	1.0	1.7	0.15
Infection due to cardiac device	0	310 (0.4)	N/A	0	0.4	N/A
Hemorrhage due to cardiac device	0	250 (0.3)	N/A	0	0.4	N/A
Pain due to cardiac device	0	137 (0.2)	N/A	0	0.2	N/A
Stenosis due to cardiac device	< 11	92 (0.12)	0.26	0.3	0.2	0.44
Pocket complications	N/A	434 (0.6)	N/A	N/A	N/A	N/A
Other complications						
Postprocedural hematoma	< 11	166 (0.2)	< 0.01	0.7	0.2	< 0.01
Postprocedural hemorrhage	< 11	69 (0.1)	< 0.01	0.8	0.1	< 0.01
Pericarditis	< 11	564 (0.7)	0.15	1.2	0.8	0.24
Acute myocardial infarction	< 11	88 (0.1)	0.03	0.4	0.2	0.33
Intraoperative cardiac arrest	< 11	101 (0.1)	0.99	0.1	0.1	0.96
Bleeding or failure of vascular closure device requiring intervention	< 11	92 (0.1)	< 0.01	0.8	0.2	< 0.01
Hemothorax	0	< 11	N/A	0	0	N/A
Pneumothorax	N/A	905 (1.2)	N/A	N/A	N/A	N/A

*Note:* To comply with the CMS cell size suppression policy, a cell containing a value of 1–10 cannot be reported directly; therefore, “< 11” is used to display a value of 1–10.

### 3.3 Chronic Complications

**Figure 2:**
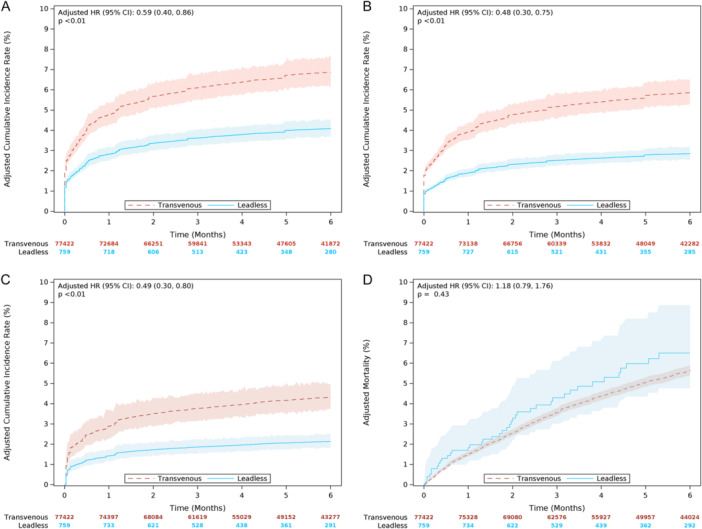
(A) 6‐Month complication rates. Hazard ratio (HR) and 95% confidence interval (CI) were based on adjusted Fine‐Gray competing risk models. Patients with an AVEIR DR leadless pacemaker (leadless) had a significantly lower 6‐month complication rate compared to patients with a dual‐chamber transvenous pacemaker. (B) 6‐Month device‐related complication rates. HR and 95% confidence interval (CI) were based on adjusted Fine‐Gray competing risk models. Patients with an AVEIR DR leadless pacemaker (leadless) had a significantly lower device‐related complication rate compared to patients with a dual‐chamber transvenous pacemaker. (C) Device reintervention (with upgrade). HR and 95% confidence interval (CI) were based on adjusted Fine‐Gray competing risk models. Patients with an AVEIR DR leadless pacemaker (leadless) had a significantly lower device reintervention rate compared to patients with a dual‐chamber transvenous pacemaker. (D) All‐cause mortality. HR and 95% confidence interval (CI) were based on adjusted Cox proportional hazard models. Patients with an AVEIR DR leadless pacemaker (leadless) had a comparable mortality rate compared to patients with a dual‐chamber transvenous pacemaker.

Compared to TP, AVEIR DR LP's overall chronic complication rate was significantly lower before and after adjustment. After adjustment, there was a significant 41% lower hazard of overall chronic complications for AVEIR DR LP compared to TP (6‐month adjusted rate 4.1% vs. 6.9%; hazard ratio [HR] 0.59, 95% CI 0.40 to 0.86, *p* < 0.01). Time to event is shown in Figure 2A. Adjusted and unadjusted overall and individual chronic complication rates at 6 months are shown in Table 3. AVEIR DR LP had 52% lower hazard of device‐related complications compared to TP as shown in Figure 2B (6‐month adjusted rate 2.8% vs. 5.9%; HR 0.48, 95% CI 0.30 to 0.75, *p* < 0.01).

**Table 3:** Unadjusted and adjusted 6‐month complication rates.

	Unadjusted			Adjusted		
	AVEIR DR (*N* = 759)%	Transvenous dual‐chamber pacemaker (*N* = 77 422)%	*p* value	AVEIR DR (*N* = 759)%	Transvenous dual‐chamber pacemaker (*N* = 77 422)%	*p* value
Overall complication	4.2%	6.6%	0.02	4.1%	6.9%	< 0.01
Individual complications						
Embolism and thrombosis						
Thrombosis due to cardiac device	0.0	0.9	N/A	0.0	0.1	N/A
Embolism due to cardiac device	0.2	0.01	< 0.01	0.1	0.0	N/A
Device‐related complication	2.9	5.7	< 0.01	2.8	5.9	< 0.01
Device malfunction	1.5	2.2	0.18	1.4	2.2	0.20
Device dislodgement	1.3	2.3	0.08	1.2	2.3	0.06
Infection due to cardiac device	0.1	1.0	0.06	0.2	1.1	0.06
Hemorrhage due to cardiac device	0.0	0.4	N/A	0.0	0.4	N/A
Pain due to cardiac device	0.0	0.3	N/A	0.0	0.3	N/A
Stenosis due to cardiac device	0.5	0.2	0.25	0.5	0.3	0.32
Pocket complication	N/A	0.9	N/A	N/A	N/A	N/A
Other complications						
Pericarditis	1.3	1.2	0.71	1.2	1.2	0.94
Hemothorax	0.0	0.01	N/A	0.0	0.01	N/A

### 3.4 Device Reintervention

Overall device reintervention rates at 6 months, which included device revisions, explants, replacements, or upgrades are shown in Figure 2C. AVEIR DR LP had 51% lower hazard of reinterventions compared to TP (6‐month adjusted rate 2.1% vs. 4.3%; HR 0.49, 95% CI 0.30 to 0.80, *p* < 0.01). Not including upgrades, AVEIR DR LP had 45% lower hazard of reinterventions compared to TP (6‐month adjusted rate 2.1% vs. 3.8%; HR 0.55, 95% CI 0.34 to 0.89, *p* = 0.02). The most common reason for reintervention was for lead revision in the TP group, and LP removal in the AVEIR DR LP group. Other individual device reinterventions are illustrated in Supporting Information S1: Table S8.

### 3.5 All‐Cause Mortality and Heart Failure Hospitalizations

There was no difference in all‐cause mortality, both at 30‐days (30‐day adjusted rate 1.8% vs. 1.5%; HR 1.21, 95% CI 0.72 to 2.02, *p* = 0.47) and 6 months (6‐month adjusted rate 6.6% vs. 5.6%; HR 1.18, 95% CI 0.79 to 1.76, *p* = 0.43) between AVEIR DR LP and TP (Figure 2D). On the day of implant, there were zero deaths among the AVEIR DR LP cohort and 25 deaths among the TP cohort. There was no difference in heart failure hospitalizations at 6 months (6‐month adjusted rate 3.8% vs. 4.1%; HR 0.91, 95% CI 0.60 to 1.37, *p* = 0.65) between AVEIR DR LP and TP.

## 4 Discussion

The present real‐world study compares complications and mortality between Medicare beneficiaries with AVEIR DR LP versus dual‐chamber TP de novo implantations. Patients with AVEIR DR LP system had significantly higher comorbidity burden compared to those with TPs and experienced a similar rate of overall acute complications, yet 50% lower device‐related complications within 30 days post‐implant. Over a longer‐term follow‐up of 6 months, the LP was associated with a 41% lower risk of overall chronic complications, 52% lower device‐related complications, and 51% lower device reinterventions. All‐cause mortality and heart failure hospitalizations were comparable between the groups, suggesting a favorable safety profile for the leadless system even in a more complex patient population. Collectively, these findings suggest that while early complication risks are comparable, leadless pacing provides a meaningful reduction in mid‐term adverse events.

AVEIR DR LP implantation occurred more frequently in the inpatient setting and in a sicker population. Operators may have preferentially selected LPs for patients with complex clinical histories, such as ESRD or recent infections, to avoid the specific risks associated with TPs. In patients with ESRD, particularly those on dialysis, venous access required for transvenous leads is often compromised due to fistulas or central venous stenosis/occlusion. LPs are associated with a lower rate of complications and better survival compared to TPs in hemodialysis patients [9, 10, 11]. Similarly, for patients with recent infection or those at high risk, the LP is advantageous as it eliminates the need for a surgical pocket, a common site of device‐related infections [12]. Furthermore, employing LPs can preserve the venous access site for future dialysis use. Additionally, frail patients can benefit from a lack of pocket and a potential reduced risk of infection.

Previous studies showed that patients implanted with a VVI‐LP had lower complications compared to patients implanted with a transvenous VVI pacemaker, which is consistent with our findings [13, 14, 15]. A recent publication from the Micra AV CED study also showed that compared to patients implanted with dual‐chamber TP, patients with a Micra AV pacemaker (Medtronic, Minneapolis, Minnesota) had 46% fewer chronic complications and 38% fewer device‐related reinterventions at 2 years [16, 17]. A majority of TP complications were associated with the subcutaneous device pocket and transvenous leads. LPs were associated with significantly fewer device‐related complications compared with transvenous systems, a finding consistent across both acute and chronic follow‐up. These results are in line with our findings, even with the AVEIR DR LP's two distinct implants. This difference is largely explained by the elimination of leads and subcutaneous pockets. Transvenous systems are vulnerable to lead fracture, insulation failure, dislodgement, venous obstruction, and pocket‐related issues such as hematoma, infection, and erosion. In contrast, LPs are self‐contained devices implanted directly within the cardiac chamber, thereby avoiding these structural failure points and sources of infection. Although LPs require a larger introducer sheath for delivery and active fixation, once implanted, their long‐term mechanical stability and lack of indwelling transvenous hardware translate into a substantially lower burden of device‐related complications [3, 13].

Device‐related extracardiac hemorrhagic complications were less common with LPs compared to TP systems. The absence of a generator pocket eliminates one of the most frequent sites of bleeding and hematoma formation after transvenous implantation; venous access and resumption of anticoagulation also contribute to hemorrhagic risk. In LP systems, increased procedure‐related bleeding risk arises from femoral venous access and is associated with occasional vascular closure device failure or local bleeding; however, this is generally less clinically significant than pocket‐related hematomas. The reduction in device‐related bleeding complications occurred despite LPs being implanted in a sicker population, with a higher prevalence of ESRD, dialysis dependence, and atrial fibrillation—all factors associated with increased risk of bleeding due to platelet dysfunction and increased anticoagulation use. This suggests that the structural advantages of avoiding a pocket outweigh the increased baseline bleeding risk in this patient group [1, 13].

Pericardial effusion and cardiac perforation were observed more frequently with AVEIR DR LPs than with TP systems (30‐day adjusted rate 1.2% vs. 0.6%; *p* = 0.047). Even though the Micra AV has a single device and Aveir DR has two separate devices, the Micra AV CED study also showed an increased rate of pericardial effusion and cardiac perforation compared to dual‐chamber TPs (30‐day adjusted rate 1.4% vs. 0.8%; *p* < 0.0001) [18]. This was also observed in the Micra VR CED study (30‐day adjusted rate 0.8% vs. 0.4%; *p* = 0.004) [13] but not in the recently published AVEIR VR CED study, where the rates were similar between LPs and TPs (30‐day adjusted rate 0.4% vs. 0.3%; *p* = 0.45) [15]. The higher perforation rate among Micra may be due to a different fixation mechanism and implantation technique. Micra relies on tines for fixation, rather than a helix mechanism. Micra also requires more forward pressure to deploy than AVEIR. For the tine fixation LP, device repositionings have been associated with increased risk of perforation [19]. The mapping capability of the newer helix fixation may lead to reduced need and number of device repositions [3]. LPs have a much larger diameter than TP leads; it has therefore been observed that LP perforations are more likely to have serious clinical repercussions compared with TP perforations [20, 21]. It has been postulated that a significant fraction of TP perforations go unrecognized clinically, while most LP perforations are more readily diagnosed [22]. In addition, LP implantation is sometimes performed with intracardiac echocardiography (ICE) imaging, which might bias toward detection and reporting of otherwise clinically insignificant pericardial effusions related to implant.

The dual‐device architecture of AVEIR DR LP raises theoretical concerns about cumulative procedural risks, given that two devices must be implanted. Our findings suggest that the longer‐term clinical benefit of avoiding leads and pockets outweighs this consideration, as evidenced by fewer chronic complications and lower reintervention rates. Over time, this may also translate into economic benefits through reduced need for repeat procedures. However, compared to a single‐device system, long‐term follow‐up may involve a higher number of device replacements due to differences in battery longevity between the two devices or the need for a two‐device replacement procedure. Improvements in technology that extend battery life may address this issue. Importantly, the dual‐device design of AVEIR DR LP, with i2i communication and atrial pacing and sensing, extends leadless pacing to a much larger cohort of patients. These results align with early reports which demonstrated reliable AV synchrony and favorable electrical performance [3, 13].

### 4.1 Limitations

Although administrative databases are increasingly used for clinical research, such studies are potentially limited by their reliance on administrative Medicare claims data, which may be prone to miscoding and underreporting of complications. Follow‐up was restricted to 6 months, and longer‐term outcomes such as device retrieval, replacement, and battery performance remain to be determined. Although propensity score overlap weighting was applied, residual confounding from unmeasured factors cannot be excluded. One such confounder may be operator experience. As a new device, AVEIR DR LPs were initially implanted by a select group of high‐volume operators. As these operators and others become more experienced with implanting AVEIR DR LP, complication rates may further reduce over time. Importantly, the analysis was restricted to Medicare FFS beneficiaries, a population that by definition includes almost exclusively patients aged 65 years or older and with a greater comorbidity burden than the general pacing population; as such, these results may not be generalized to younger patients, those with commercial insurance, or international populations where patient selection, operator experience, and healthcare delivery differ. Broader studies in more diverse populations will be necessary to confirm the safety and effectiveness of dual‐chamber leadless pacing across all practice settings, and ongoing registry and trial data outside of Medicare will be essential to address these gaps.

## 5 Conclusion

AVEIR DR leadless dual‐chamber pacemaker provides comparable short‐term safety and significantly fewer device‐related complications and reinterventions at 6 months compared with traditional transvenous systems, despite implantation in a sicker population in this real‐world analysis. These findings support the expanding role of leadless pacing in routine clinical practice and highlight the importance of continued long‐term surveillance through the CMS CED framework.

## Conflicts of Interest

Monica Lo: Abbott (consultant, advisory board); Marcin Kowalski: Abbott (advisor, speaker); Anne Kroman: Abbott (consultant, speaker); Stanislav Weiner: Consultant ABT, BSC, JNJ, MDT, Research: Bayer, BSC, Impulse Dynamics, MDT, Recor Medical, V‐Wave; Jennifer M. Joseph: Abbott; Yajing Hu: Abbott; Yelena Nabutovsky: Abbott; Leonard Ganz: Abbott; Mohammed Mortada: Abbott (advisor, speaker). The other authors declare no conflicts of interest.

## Supporting information

Aveir_DR_vs_Transvenous_supplement.
