# Supplementary material for: Leadless Versus Transvenous Dual‐Chamber Pacemakers: Real‐World Evidence From AVEIR DR Coverage With Evidence Development Study
Source: J Cardiovasc Electrophysiol. 2026 Jan 16;37(3):544–53. doi: 10.1111/jce.70255 (PMC12980453; doi:10.1111/jce.70255)
Supplement: Supplementary file 1 — Aveir_DR_vs_Transvenous_supplement. [file JCE-37-544-s001.docx]

**Leadless vs. Transvenous Dual-Chamber Pacemakers: Real-World Evidence from AVEIR DR Coverage with Evidence Development Study**

**Supplement**

**S1. AVEIR DR and dual-chamber transvenous pacemaker device implant codes**

| *CPT and ICD-10 Procedure Codes* | |
| --- | --- |
| AVEIR DR leadless pacemaker system implant | 0795T, 02HK3NZ AND 02H63NZ, X2H63V9 AND X2HK3V9 |
| Dual-chamber transvenous pacemaker system implant | 33208, 33213, 0JH606Z, 0JH636Z, 0JH806Z, 0JH836Z |

**S2. Cardiac Implantable Electronic Device (CIED) procedure codes**

| *CPT and ICD-10 Procedure Codes* |
| --- |
| CIED implant, including leadless single-chamber pacemaker, transvenous single-chamber pacemaker, dual-chamber leadless pacemaker, dual-chamber transvenous pacemaker, implantable cardioverter defibrillator (ICD), or cardiac resynchronization therapy pacemaker (CRT-P) or defibrillator (CRT-D) |
| "3781","3782","0JH604Z","0JH605Z","0JH634Z","0JH635Z","33207","0JH805Z","0JH835Z","0JH834Z","3783","0JH606Z","0JH636Z","33208","0JH806Z","0JH836Z","0051","0054","0JH609Z","0JH639Z","0JH809Z","0JH839Z","33263","33249","33264","0050","0053","0JH607Z","0JH637Z","0JH807Z","0JH837Z","33214","33229","3780","3783","3787","0JH60PZ","0JH63PZ","0JH80PZ","0JH83PZ","33213","33228","3794","3796","3798","0JH608Z","0JH638Z","0JH808Z","0JH838Z","33221","33230","33240","33262","33270","3779","3781","3782","3785","3786","0JH804Z","33206","33212","33227","0387T","02HK3NZ","02HK0NZ","02HK4NZ","33224","33226","33231","33233","33222","33215","33216","33217","33218","33220","33234","33235","33241","33223","02HK4JZ","02HK3JZ","02HK0JZ","0JPT0PZ","0JPT3PZ","02WA0MZ","02WA3MZ","02WA4MZ","0JWT0PZ","0JWT3PZ","02PA3NZ","02WA3NZ","02H43MZ","02H63KZ","02HK3KZ","02HN0KZ","02HN4KZ","02HK4KZ","02HK0KZ","02H60KZ","02H64KZ","02HL4JZ","02HL3JZ","02HL0JZ","02H44JZ","02H43JZ","02H40JZ","02HL4KZ","02HL3KZ","02HL0KZ","02H44KZ","02H43KZ","02H40KZ","02H73KZ","02H70KZ","02H74KZ","33274","33275","93279","93288","93286","93280","93281","93293","93294","93296","93282","93283","93284","93289","93287","93295","02H63NZ","0JH60PZ","0571T","33225","93743","93744","93731","93734","93741","93640","93641","02HK3DZ","0388T","02PA3DZ","02WA3DZ","0JH805Z","02HK0MZ","02HK3JZ","02HK3MZ","02HK4JZ","02HK4MZ","02HN0JZ","02HN0MZ","02HN3JZ","02HN3MZ","02HN4JZ","02HN4MZ","33238","02PA0MZ","02PA3MZ","02PA4MZ","02PAXMZ","3780","3785","3786","3787","93260","93261","93644","0389T","0390T","0391T","93724","93732","93733","93735","93736","33273","33243","33244","33272","33271","G0448","3771","3772","3770","3773","3776","3775","3777","3789","8945","8946","8947","8948","8949","4B02XSZ","4B02XTZ","3797","02H60JZ","02H63JZ","02H64JZ","02H60MZ","02H63MZ","02H64MZ","02H40MZ","02H44MZ","02HL0MZ","02HL3MZ","02HL4MZ","0052","3795","3796","3794","3796","3798","0JH63FZ","0JH60FZ","0319T","0320T","0321T","0324T","0JPT0PZ","02HN3KZ","02H70JZ","02H73JZ","02H74JZ","02H70MZ","02H73MZ","02H74MZ", “0795T”, “0796T”, “X2H63V9”, “X2HK3V9”, “0823T” |

**S3. Acute complication codes**

Acute complications were identified by the presence of one or more of the diagnosis codes listed below (occurring within 30 days of the implant).

| **Event** | **Code** |
| --- | --- |
| *ICD-10 Diagnosis Codes* | |
| Acute myocardial infarction during cardiac surgery | (I97.190, I97.790) and (I21.01, I21.02, I21.09, I21.11, I21.19, I21.21, I21.29, I21.3, I21.4, I21.9, I21.A1, I21.A9) |
| Arteriovenous fistula | I77.0 |
| Cardiac perforation | I97.51 |
| Cardiac tamponade | I31.4 |
| Deep vein thrombosis | I82.401, I82.402, I82.403, I82.409, I82.411, I82.412, I82.413, I82.419, I82.421, I82.422, I82.423, I82.429, I82.4Y1, I82.4Y2, I82.4Y3, I82.4Y9, I82.4Z1, I82.4Z2, I82.4Z3, I82.4Z9, I82.621, I82.622, I82.623, I82.629, I82.A11, I82.A12, I82.A13, I82.A19 |
| Device dislodgement or displacement | T82.12++ |
| Embolism due to cardiac prosthetic devices, implants, and grafts | T82.817+ |
| Hematoma | I97.638 |
| Hemorrhage | I97.618, T82.837+ |
| Hemothorax | (J95.62 or J95.831) plus J94.2, J95.72 plus J94.2 |
| Infection | T82.7+++ |
| Intraoperative cardiac arrest | I97.710 or I97.120 |
| Pain due to cardiac prosthetic device, implant and grafts | T82.847+ |
| Pericardial effusion | I97.51 and (I30.9, I31.3) |
| Pericarditis | I30.9 or I31.9 |
| Pneumothorax | J95.811 or J95.812 |
| Pulmonary embolism | I26.01, I26.02, I26.09, I26.90, I26.92, I26.99 |
| Stenosis due to cardiac prosthetic device, implant and grafts | T82.857+ |
| Thrombosis due to cardiac prosthetic devices, implants, and grafts | T82.867+ |
| Vascular complication – bleeding or failure of vascular closure device requiring intervention | I97.418 or I97.618 |
| Vascular pseudoaneurysm | I72.4 |
| Other cardiac device malfunction | T82.11++, T82.19++ |
| Pocket complication | T82.897+ |

**S4. Chronic complication codes**

Chronic complications were identified by the presence of one or more of the diagnosis codes listed below (occurring within 6 months of the implant).

| **Event** | **Code** |
| --- | --- |
| *ICD-10 Diagnosis Codes* | |
| Device dislodgement or displacement | T82.12++ |
| Embolism due to cardiac prosthetic devices, implants, and grafts | T82.817+ |
| Hemorrhage | T82.837+ |
| Hemothorax | (J95.62 or J95.831) plus J94.2 J95.72 plus J94.2 |
| Infection | T82.7+++ |
| Pain due to cardiac prosthetic device, implant and grafts | T82.847+ |
| Pericarditis | I30.9 or I31.9 |
| Stenosis due to cardiac prosthetic device, implant and grafts | T82.857+ |
| Thrombosis due to cardiac prosthetic devices, implants, and grafts | T82.867+ |
| Other cardiac device malfunction | T82.11++, T82.19++ |
| Pocket complication | T82.897+ |

**S5. Device-related reintervention codes**

Device-related reinterventions were identified by the presence of a procedure code that indicates a device explant or a device revision or a single or dual-chamber pacemaker (leadless or transvenous) implant or an upgrade to a cardiac resynchronization therapy device or implantable cardioverter defibrillator.

| **Event** | **Code** |
| --- | --- |
| *CPT and ICD-10 Procedure Codes* | |
| Device explant; device revision; single- or dual-chamber pacemaker implant (leadless or transvenous) | Leadless pacemakers: 33274,33208, 33206, 02HK3NZ, 02H63NZ, 33275, 02PA3NZ, 02WA3NZ, 33207, 0JH604Z, 0JH634Z, 0JH804Z, 0JH834Z, 0JH605Z, 0JH635Z, 0JH805Z, 0JH835Z, 0JH606Z, 0JH636Z, 0JH806Z, 0JH836Z, 0795T, 0798T, 0799T, 0800T, 0801T, 0802T, 0803T, X2H63V9, X2HK3V9  Transvenous pacemakers: 33206, 33207, 33208, 0JH604Z, 0JH634Z, 0JH804Z, 0JH834Z, 0JH605Z, 0JH635Z, 0JH805Z, 0JH835Z, 0JH606Z, 0JH636Z, 0JH806Z, 02HK0JZ, 02HK0MZ, 02HK3JZ, 02HK3MZ, 02HK4JZ, 02H60JZ, 02H63JZ, 02H64JZ, 02H60MZ, 02H63MZ, 02H64MZ, 02HK4MZ, 02HN0JZ, 02HN0MZ, 02HN3JZ, 02HN3MZ, 02HN4JZ, 02HN4MZ, 33212, 33213, 33215, 33216, 33217, 33218, 02WA0MZ, 02WA3MZ, 02WA4MZ, 33220, 33228, 33234, 33235, 33238, 02PA0MZ, 02PA3MZ, 02PA4MZ, 02PAXMZ, 0JWT0PZ, 0JWT3PZ, 33233, 0JPT0PZ, 0JPT3PZ, 33222, 02HK3NZ, 0JH836Z, 02H63NZ, 33274, 0795T, X2H63V9, X2HK3V9 |
| Upgrade | 33221, 33224, 33225, 33229, 33230, 33231, 33240, 33249, 33262, 33263, 33264, 33270, 0571T, 0JH607Z, 0JH609Z, 0JH637Z, 0JH639Z, 0JH807Z, 0JH809Z, 0JH837Z, 0JH839Z, 0JH608Z, 0JH638Z, 0JH808Z, 0JH838Z, 0JH60PZ, 0JH63PZ, 0JH80PZ, 0JH83PZ |

**S6. Clinical characteristics codes**

| **Clinical Characteristics** | **Code** |
| --- | --- |
| Atrial and Ventricular Arrhythmias |  |
| Atrial Fibrillation | Any codes in the following ranges: I48.0-I48.2 or I48.91 |
| Atrial Flutter | Any codes in the following ranges: I48.3-I48.4 or I48.92 |
| History of Supraventricular Tachycardia | I47.1 |
| History of Ventricular Arrythmia | Any of the following codes: Z86.74 or I47.0 or I47.2 or I49.01 or I49.02 or I49.3 |
|  | - Myocardial infarction: I21.x, I22.x, I25.2 - Congestive heart failure: I09.9, I11.0, I13.0, I13.2, I25.5, I42.0, I42.5 - I42.9, I43.x, I50.x, P29.0 - Peripheral vascular disease: I70.x, I71.x, I73.1, I73.8, I73.9, I77.1, I79.0, I79.2, K55.1, K55.8, K55.9, Z95.8, Z95.9 - Cerebrovascular disease: G45.x, G46.x, H34.0, I60.x - I69.x - Dementia: F00.x - F03.x, F05.1, G30.x, G31.1 - Chronic pulmonary disease: I27.8, I27.9, J40.x - J47.x, J60.x - J67.x, J68.4, J70.1, J70.3 - Rheumatic disease: M05.x, M06.x, M31.5, M32.x - M34.x, M35.1, M35.3, M36.0 - Peptic ulcer disease: K25.x - K28.x - Mild liver disease: B18.x, K70.0 - K70.3, K70.9, K71.3 - K71.5, K71.7, K73.x, K74.x, K76.0, K76.2 - K76.4, K76.8, K76.9, Z94.4 - Diabetes without chronic complication: E10.0, E10.1, E10.6, E10.8, E10.9, E11.0, E11.1, E11.6, E11.8, E11.9, E12.0, E12.1, E12.6, E12.8, E12.9, E13.0, E13.1, E13.6, E13.8, E13.9, E14.0, E14.1, E14.6, E14.8, E14.9 - Diabetes with chronic complication: E10.2 - E10.5, E10.7, E11.2 - E11.5, E11.7, E12.2 - E12.5, E12.7, E13.2 - E13.5, E13.7, E14.2 - E14.5, E14.7 - Hemiplegia or paraplegia: G04.1, G11.4, G80.1, G80.2, G81.x, G82.x, G83.0 - G83.4, G83.9 - Renal disease: I12.0, I13.1, N03.2 - N03.7, N05.2 - N05.7, N18.x, N19.x, N25.0, Z49.0 - Z49.2, Z94.0, Z99.2 - Any malignancy, including lymphoma and leukemia, except malignant neoplasm of skin: C00.x -C26.x, C30.x - C34.x, C37.x - C41.x, C43.x, C45.x - C58.x, C60.x - C76.x, C81.x - C85.x, C88.x, C90.x - C97.x - Moderate or severe liver disease: I85.0, I85.9, I86.4, I98.2, K70.4, K71.1, K72.1, K72.9, K76.5, K76.6, K76.7 - Metastatic solid tumor: C77.x - C80.x - AIDS/HIV: B20.x - B22.x, B24.x |
| Chronic Obstructive Pulmonary Disease | Any code in the following ranges: J43.0-J43.9 or J44.0-J44.9 or J47.0-J47.9 or J60-J63.6 |
| Coronary Artery Disease | Any code in the following ranges: I25.10-I25.119 or I25.700-I25.739 or I25.790-I25.799 or I25.810 or I25.750-I75.769 or I25.811-I25.812 or I25.82-I25.84 |
| Diabetes | Any code in the following ranges: E08.00-E08.9 or E09.00-E09.9 or E10.10-E10.9 or E11.00-E11.9 or E13.00-E13.9) |
| Heart Failure | Any code in the following ranges: I09.81 or I11.0 or I13.0 or I13.2 or I50.20-I50.9 or I97.130-I97.131 |
| Hyperlipidemia | E78.1-E78.5 |
| Hypertension | Any code in the following ranges: I10 or I11.0-I11.9 or I12.0-I12.9 or I13.0-I13.2 or I15.0-I15.9 or I16.0-I16.9 or I97.3 |
| Peripheral Vascular Disease | Any code in the following ranges: I70.201-I70.299 or I70.301-I70.799 or I73.00-I73.9 |
| Prior Cardiovascular Events And Procedures within 1 year prior to implant |  |
| Prior Coronary Artery Bypass Graft | Any code in the following ranges: Z95.1 or T82.211A-T82.218S or I25.700-I25.739 or I25.790-I25.799 or I25.810 |
| Prior Acute Myocardial Infarction | Any code in the following ranges: I25.2 or I21.01-I21.4 |
| Prior Percutaneous Coronary Intervention | Any code in the following ranges: Z95.5 or Z98.61 or T82.855A-T82.855S |
| Concomitant Atrial Ablation | 93650 or 93653 or 93656 or 93657 or 02583ZZ + (I48.0-I48.2 or I48.91) |
| Concomitant Transcatheter Aortic Valve Replacement | 33361or 33362 or 33363 or 33364 or 33365 or 33366 or 02RF38Z or 02RF38H |
| Prior Transcatheter Aortic Valve Replacement | 33361or 33362 or 33363 or 33364 or 33365 or 33366 or 02RF38Z or 02RF38H |
| Renal Disease | Any code in the following ranges: K76.7 or N17.0-N17.9 or N18.1-N18.9 or N19 or N28.9 or N99.0 or R39.2 |
| End Stage Kidney Disease | N18.5, N18.6, I12.0, I13.11 |
| Dialysis Dependence | Z49.01, Z49.02, Z49.31, Z49.32, Z91.15, Z99.2,   \| '031509D', '031509F', '031509V', '03150AD', '03150AF', '03150AV', '03150JD', '03150JF', '03150JV', '03150KD', '03150KF', '03150KV', '03150ZD', '03150ZF', '03150ZV', '031609D', '031609F', '031609V', '03160AD', '03160AF', '03160AV', '03160JD', '03160JF', '03160JV', '03160KD', '03160KF', '03160KV', '03160ZD', '03160ZF', '03160ZV', '031709D', '031709F', '031709V', '03170AD', '03170AF', '03170AV', '03170JD', '03170JF', '03170JV', '03170KD', '03170KF', '03170KV', '03170ZD', '03170ZF', '03170ZV', '031809D', '031809F', '031809V', '03180AD', '03180AF', '03180AV', '03180JD', '03180JF', '03180JV', '03180KD', '03180KF', '03180KV', '03180ZD', '03180ZF', '03180ZV', '031909F', '03190AF', '03190JF', '03190KF', '03190ZF', '031A09F', '031A0AF', '031A0JF', '031A0KF', '031A0ZF', '031B09F', '031B0AF', '031B0JF', '031B0KF', '031B0ZF', '031C09F', '031C0AF', '031C0JF', '031C0KF', '031C0ZF', '041C09D', '041C09F', '041C0AD', '041C0AF', '041C0JD', '041C0JF', '041C0KD', '041C0KF', '041C0ZD', '041C0ZF', '041K09S', '041K0AS', '041K0JS', '041K0KS', '041K0ZS', '041L09S', '041L0AS', '041L0JS', '041L0KS', '041L0ZS' \| \| --- \| \|  \| |
| Tricuspid Valve Disease | Any code in the following ranges: I07.I-I07.9 or I08.1-I08.3 or I36.0-I36.9 or Q22.4 or Q22.8-Q22.9 |
| COVID-19 | U07.1, B97.29 |

**S7. 30-Day Complications– Time to Event**

Patients with an AVEIR DR leadless pacemaker (Leadless) had a comparable acute complication rate compared to patients with a dual-chamber transvenous pacemaker.


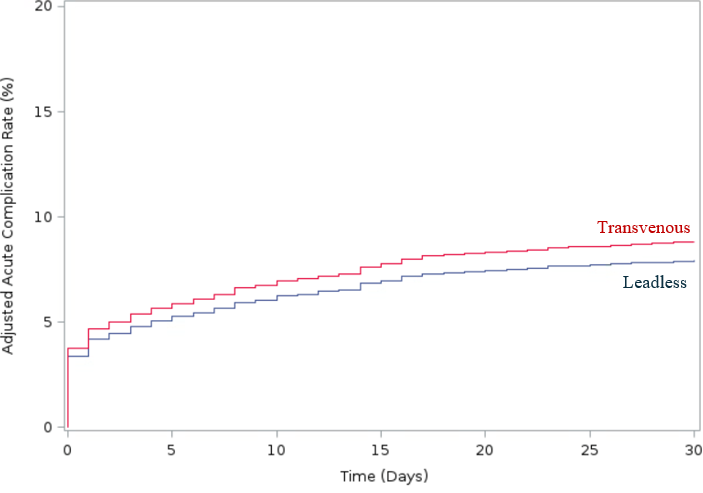


**S8. Rates of Individual Reinterventions**

| **AVEIR DR**  **(N = 759)** | |  | **Dual-chamber Transvenous**  **(N = 77,422)** | |
| --- | --- | --- | --- | --- |
|  | N |  |  | N (%) |
| Leadless implant or replacement | <11^*^ |  | Replacement with Leadless | 110 (0.1) |
| Leadless removal | <11^*^ |  | Device replacement | 439 (0.6) |
| Leadless revision | <11^*^ |  | Device removal | 286 (0.4) |
| Transvenous implant | <11^*^ |  | Device revision | 137 (0.2) |
|  |  |  | Lead-related reintervention | 1832 (2.4) |
| Upgrade to CRT or ICD | 0 |  | Upgrade to CRT or ICD | 307 (0.4) |

^*^To comply with the Centers for Medicare and Medicaid Services (CMS) cell size suppression policy, a cell containing a value of 1 to 10 cannot be reported directly; therefore, '< 11' is used to display a value of 1 to 10.

Individual device reintervention rates were not compared between treatment groups and therefore are not adjusted.
